# Whole-exome sequencing identifies a de novo *PDE3A* variant causing autosomal dominant hypertension with brachydactyly type E syndrome: a case report

**DOI:** 10.1186/s12881-020-01077-z

**Published:** 2020-07-06

**Authors:** Xianqing Li, Zongzhe Li, Peng Chen, Yan Wang, Dao Wen Wang, Dao Wu Wang

**Affiliations:** 1 Division of Cardiology, Department of Internal Medicine and Genetic Diagnosis Centre, Tongji Hospital, Tongji Medical College, Huazhong University of Science and Technology, Wuhan, China; 2 Hubei Key Laboratory of Genetics and Molecular Mechanisms of Cardiological Disorders, Huazhong University of Science and Technology, Wuhan, China; 3 State Key Laboratory of Reproductive Medicine, The Centre for Clinical Reproductive Medicine and Department of Cardiology, The First Affiliated Hospital of Nanjing Medical University, 300 Guangzhou Road, Nanjing, 210029 P. R. China

**Keywords:** Hypertension, Brachydactyly, Hypertension with brachydactyly type E syndrome, Whole-exome sequencing, Genetic diagnosis

## Abstract

**Background:**

Autosomal dominant hypertension with brachydactyly type E syndrome caused by pathogenic variants in the *PDE3A* gene was first reported in 2015. To date, there are only a few reports of this kind of syndrome. Other patients still lack a genetic diagnosis.

**Case presentation:**

Whole-exome sequencing was performed in an 18-year-old female proband with a clinical diagnosis of hypertension with brachydactyly syndrome. Quantitative real-time PCR was used to identify pathogenic copy number variations (CNVs). After bioinformatics analysis and healthy control database filtering, we revealed a heterozygous missense *PDE3A* variant (c.1346G > A, p.Gly449Asp). The variant was absent in the ExAC database and located in a highly evolutionarily conserved cluster of reported *PDE3A* pathogenic variants. Importantly, this variant was predicted to affect protein function by both SIFT (score = 0) and PolyPhen-2 (score = 1). After Sanger sequencing, the variant was determined to be absent in the healthy parents of the proband as well as 800 ethnically and geographically matched healthy controls.

**Conclusion:**

We present a report linking a de novo *PDE3A* variant to autosomal dominant hypertension with brachydactyly type E syndrome.

## Background

In 1973, Biliginturan et al. [1] reported a large no consanguineous Turkish family showing brachydactyly associated with hypertension, and the authors clinically described the phenotype of the disorder in detail for the first time. Schuster et al. [2] discovered severe autosomal dominant hypertension and brachydactyly in a unique Turkish lineage mapped to human chromosome 12p. In 2015, Maass et al. [3] identified mutations in the *PDE3A* gene at 12p12.2 in six families with hypertension with brachydactyly type E syndrome (HTNB) (OMIM: 112410). HTNB is an autosomal dominant disease characterized by brachydactyly type E (BDE) and severe hypertension [2, 4, 5]. Patients with HTNB feature salt-independent and sharply increasing blood pressure with age from childhood [4]. They usually suffer from stroke (haemorrhagic or thrombotic) if untreated, usually before the age of 50 years [6]. HTNB patients usually have a stockier build, shorter stature and rounder face than healthy persons [6, 7].

Cyclic nucleotide phosphodiesterases (PDEs) regulate intracellular signalling by hydrolysing cyclic AMP (cAMP) and cyclic GMP (cGMP) [8, 9]. They thereby play key roles in regulating the amplitude and duration of many cellular processes and, consequently, myriad biological responses in healthy persons and persons with disease [9, 10]. PDE3A, a cGMP-inhibited cAMP PDE, plays important roles in cardiovascular function by regulating vascular smooth muscle contraction and relaxation [9–11].

In 2015, Maasset al. revealed for the first time that the *PDE3A* gene at 12p12.2 is the causative gene for HTNB [3]. The authors demonstrated that gain-of-function *PDE3A* variants (p.Thr445Ala; p.Thr445Ser; p.Thr445Asn; p.Ala447Thr; p.Ala447Val; and p.Gly449Val) upregulate protein kinase A (PKA)-mediated *PDE3A* phosphorylation, therefore increasing cAMP hydrolytic activity and producing lower intercellular cAMP concentrations in vascular smooth muscle cells (VSMCs) [3]. On the one hand, the variants promoted peripheral vascular constriction and increased VSMC proliferation, thus causing hypertension; on the other hand, they regulated chondrogenesis and resulted in brachydactyly [3]. In 2016, Boda et al. identified another pathogenic variant (p.Ser446Pro) in the same cluster region in exon 4 of the *PED3A* gene causing HTNB in a Japanese family [6]. In 2018, Hauer *et al*. identified a pathogenic variant (p.Gly449Asp) by whole-exome sequencing for short stature [12].

Here, we present another report linking the *PDE3A* variant to HTNB. We identified a de novo *PDE3A* variant in the pathogenic cluster in a Han Chinese family with HTNB.

## Case presentation

The ethics committee of Tongji Hospital approved our study. Written informed consent was obtained from the patient. Our experiments followed the principles expressed in the Declaration of Helsinki.

To identify the genetic cause in the proband, total DNA was isolated from the blood of each participant using a QIAamp DNA Mini Kit (Qiagen, Germany). Then, we checked the purified DNA using electrophoresis to avoid fragmental DNA degradation and RNA pollution.

We performed WES using an Ion AmpliSeq™ Exome Panel (Hi-Q) (57.7 Mb target region) on an Ion Proton Sequencer (Life Technologies, Thermo Fisher, USA). Library construction and next-generation sequencing were performed as we previously described [13].

We initially processed the data with the Ion Torrent platform-specific software Torrent Suite v5.0.4 to generate reads and trim adapter sequences. Then, reads were aligned to the hg19/GRCh37 human reference genome (https://genome.ucsc.edu/cgi-bin/hgTracks?db=hg19&position=lastDbPos) to analyse coverage status, and the consensus on-target reads were used to call variants using recommended germline parameters. Finally, the annotation was performed with Ion Reporter 5.0 (Life Technologies, Thermo Fisher, USA). The datasets generated and/or analysed during the current study are available in the SRA - NCBI repository, the Sequence Read Archive (SRA) accession number is: SRR11974541 (https://www.ncbi.nlm.nih.gov/sra/?term=SRR11974541).

To identify reported pathogenic variants, we searched the ClinVar database (https://www.ncbi.nlm.nih.gov/clinvar/) and the HGMD database (http://www.hgmd.cf.ac.uk/ac/search.php). To filter non-pathogenic variants, we removed low-quality data (coverage less than 15-fold) and variants with a minor allele frequency (MAF) greater than 0.1% in the Exome Sequencing Project database (http://evs.gs.washington.edu/EVS/), the 1000 Genomes Project database (http://browser.1000genomes.org/) and the ExAC database (http://exac.broadinstitute.org/). We removed intronic, up/downstream, and synonymous variants. Furthermore, we filtered out the variants in our exome database of 100 healthy Chinese. Then, we predicted the impact scores of the missense variants using both PolyPhen-2 scores (http://genetics.bwh.harvard.edu/pph2/) and SIFT scores (http://sift.jcvi.org). We also evaluated their evolutionary conservation across multiple species by using phyloP scores. Finally, we filtered out variants in genes unrelated to hypertension. For WES data filtering procedures, a filtering tree illustrated the step-by-step narrowing down of candidate gene/variants for the illustrated NGS data analysis (Supplementary Fig. S1).

We performed Sanger sequencing of all identified pathogenic variants and low-coverage regions of *PDE3A* by using an Applied Biosystems 3500xl sequencer (Applied Biosystems). PCR amplification and BigDye reactions were optimized using Taq™ Hot Start enzyme (TaKaRa) with a BigDye Terminator Cycle Sequencing Kit. After validation in the patient, the potential pathogenic variant was also directly Sanger sequenced in 800 unrelated ethnically and geographically matched healthy controls. We performed Sanger sequencing-based (21 loci) paternity/maternity tests for the three family members.

The multiplex parallel AmpliSeq technique of the Ion Torrent platform allowed us to analyse CNVs directly based on the sequencing data by using the CNV workflow in Ion Reporter™ software 5.0. We performed whole-exome CNV analysis using exome sequencing data of the proband and healthy controls that we previously sequenced. All identified CNVs were validated by gDNA real-time PCR analysis for each exon. We also performed real-time PCR-based CNV analysis of each exon of the *PDE3A* gene. The primers are listed in Table 1.

**Table 1:** Primer sequences of exons of the *PDE3A* gene

Primer	Sequence
Exon 1-F	5′ TTCAGTGAAGAGGGCACCCTAT 3’
Exon 1-R	5′ GTCCGCACGATGGTGGC 3’
Exon 2&3-F	5′ GGAAGCGCTCGTCCAGAT 3’
Exon 2&3-R	5′ AAGGAAATGAGGGACACGGT 3’
Exon 4-F	5′ TGAATCCCGTCACTTCGCTC 3’
Exon 4-R	5′ GGTCTCTCCGTACTGGTGCA 3’
Exon 5&6-F	5′ GGAGCCTGCACCAGTACGG 3’
Exon 5&6-R	5′ GGAGCAGGGCGATGAAAGA 3’
Exon 7-F	5′ TGCCCCAGACCTATCCCCT 3’
Exon 7-R	5′ TTGGTGTCCGAGTGGCTACC 3’
Exon 8-F	5′ TGACTCCACCTGTTATATGTAGCAG 3’
Exon 8-R	5′ AGCCGATGCTTTCCTCAGAG 3’
Exon 9&10-F	5′ CTGAGGAAAGCATCGGCTT 3’
Exon 9&10-R	5′ AAAAGCTTCAAAGAGGCCC 3’
Exon 11&12-F	5′ GAAGACATGGGCCTCTTTGAAG 3’
Exon 11&12-R	5′ ATGTCCATGTGTAAATCCACTGTC 3’
Exon 13-F	5′ TTGGAGTTGATGGCGCTGTAT 3’
Exon 13-R	5′ ACAAGGAAACGGAAATGCTTAA 3’
Exon 14-F	5′ ACTTCGTAGCCAAATTTAATGG 3’
Exon 14-R	5′ GGCCTCTTCATCACCCTGT 3’
Exon 15-F	5′ TGATATCAATGGTCCAGCTAAATG 3’
Exon 15-R	5′ CCCACAATGTGAGAGATGAAGG 3’
Exon 16-F	5′ CACATTGTGGGGCCTCTGT 3’
Exon 16-R	5′ TTCTATGCCTGCCAACCGT 3’

To investigate the influence of the identified pathogenic variants, protein structural modelling was performed using homologous protein structure in SPDBV 4.10 online software (http://spdbv.vital-it.ch/).

Statistical analysis was performed using SPSS software version 20.0. Differences between groups were test for significance using the Pearson chi-square test, and a two-sided *P* value less than 0.05 was considered statistically significant.

**Fig. 1:**
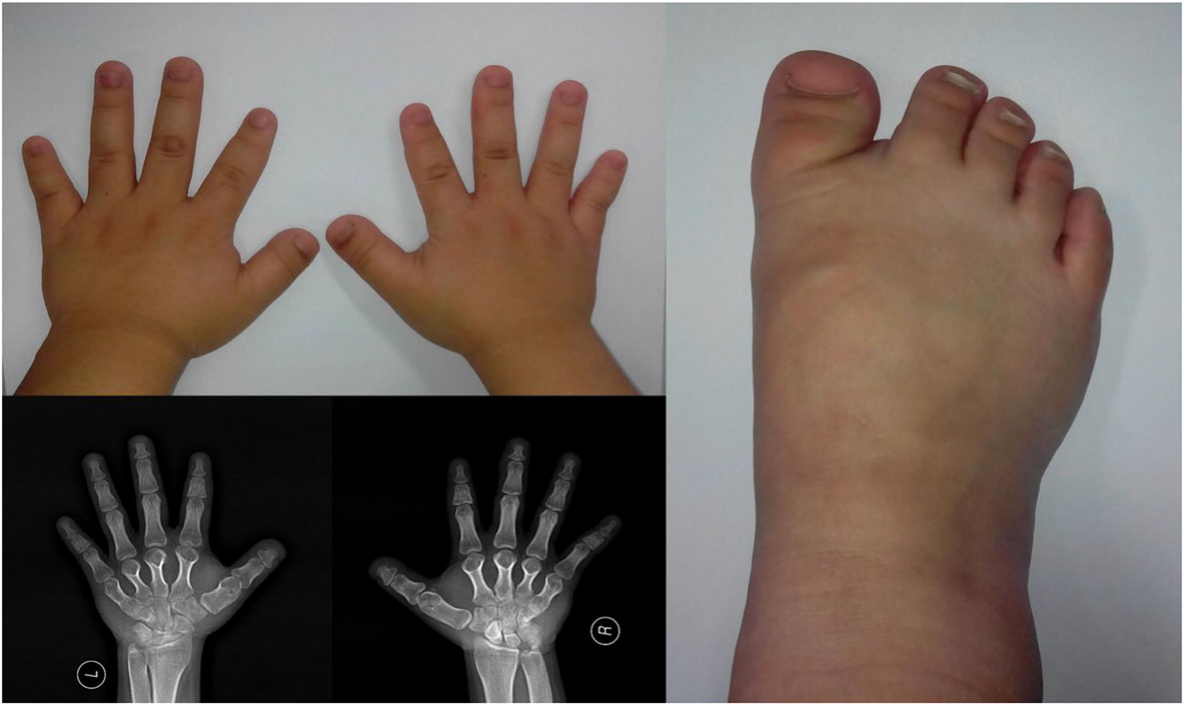
Photos and radiographic images of the patient’s hands and right foot showing short fingers and toes associated with brachydactyly type E (BDE)

An 18-year-old female of short stature (152 cm) was referred to our hospital with a two-year history of hypertension. She underwent 24-h blood pressure measurement. Her systolic pressure ranged from 150 to 220 mmHg and diastolic pressure ranged from 80 to 120 mmHg. She exhibited short fingers, short toes and normal intelligence. The radiographic images of her fingers and toes revealed BDE (Fig. 1). Renal ultrasonography and computed tomography angiography (CTA) of her renal arteries did not present obvious anomalies. Duplex tests of plasma renin activity and aldosterone, catecholamine measurements, liver and kidney functions, and electrolytes were all in the normal range. These results imply primary hypertension instead of a known cause of secondary hypertension. She underwent funduscopic examination and an echocardiogram. Both examinations presented normal results. There was no family history of hypertension or stroke. Both of her parents presented normal blood pressure (~ 130/70 mmHg) and normal stature (father: 175 cm, mother: 168 cm) and did not show obvious anomalies during physical or laboratory examinations.

By WES, we obtained an output of 34,062,121 mapped reads, 93.49% of which were on target. In conclusion, 99.54% of all 293,903 target amplicons were covered at least once, and 94.55% of amplicons were covered at least 20 times. The average base-read depth was 108-fold.

**Fig. 2:**
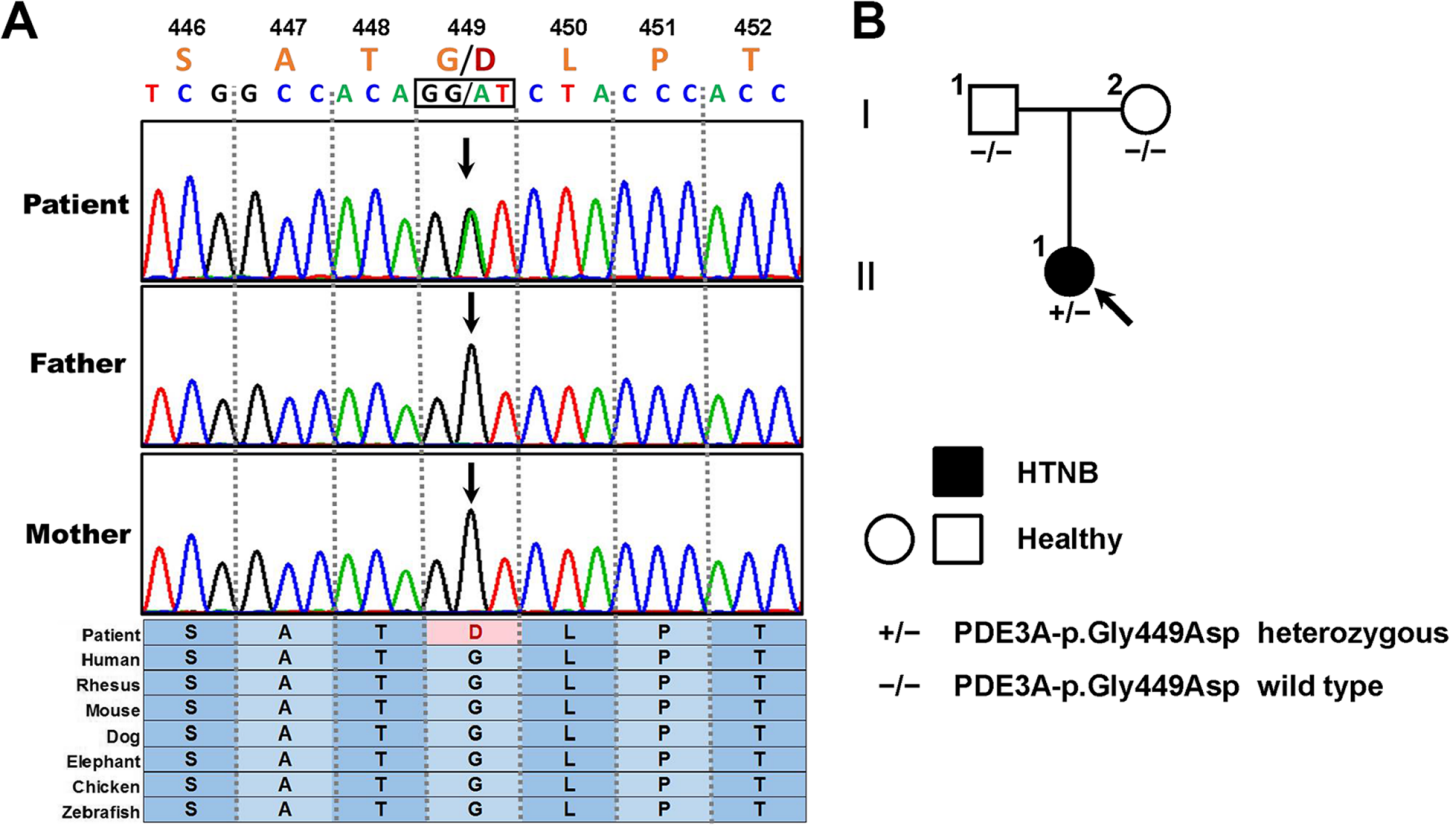
The pathogenic variant and pedigree description. **a** Sanger sequencing of the family. Coloured blocks show the evolutionary conservation of the cluster across multiple species. **b** The family tree of the HTNB pedigree. Males and females are indicated by squares and circles, respectively. The filled symbol represents the HTNB-affected individual. The arrow shows the proband. +/− represents the heterozygous *PDE3A* p.Gly449Asp variant. −/− represents the wild type

After filtering and Sanger sequencing validation, we found a heterozygous missense variant, c.1346G > A (p.Gly449Asp), in exon 4 of the *PDE3A* gene (Fig. 2a), according to the guidelines [14]. No other variants or suspicious CNVs were detected in the coding region of known hypertension-related genes. The identified variant was successfully validated in the proband by Sanger sequencing; however, it was absent in her parents, which implied a de novo inheritance mode (Fig. 2b). Furthermore, it was absent in the 800 unrelated matched healthy controls, which implied that it was not a rare SNP in Chinese. Moreover, the identified pathogenic variant was in neither the ESP database nor the ExAC database. The variant was in a highly evolutionarily conserved region across multiple species (phyloP score = 2.7). *PDE3A*-p.Gly449Asp was predicted to be a protein function-affecting variant (SIFT score = 0, PolyPhen-2 score = 1).

## Discussion and conclusions

We report a *PDE3A* variant related to HTNB. Our finding implies that the c.1346G > A (p.Gly449Asp) variant, in exon 4 of the *PDE3A* gene, may be the genetic cause in the patient of a Han Chinese family with HTNB.

**Fig. 3:**
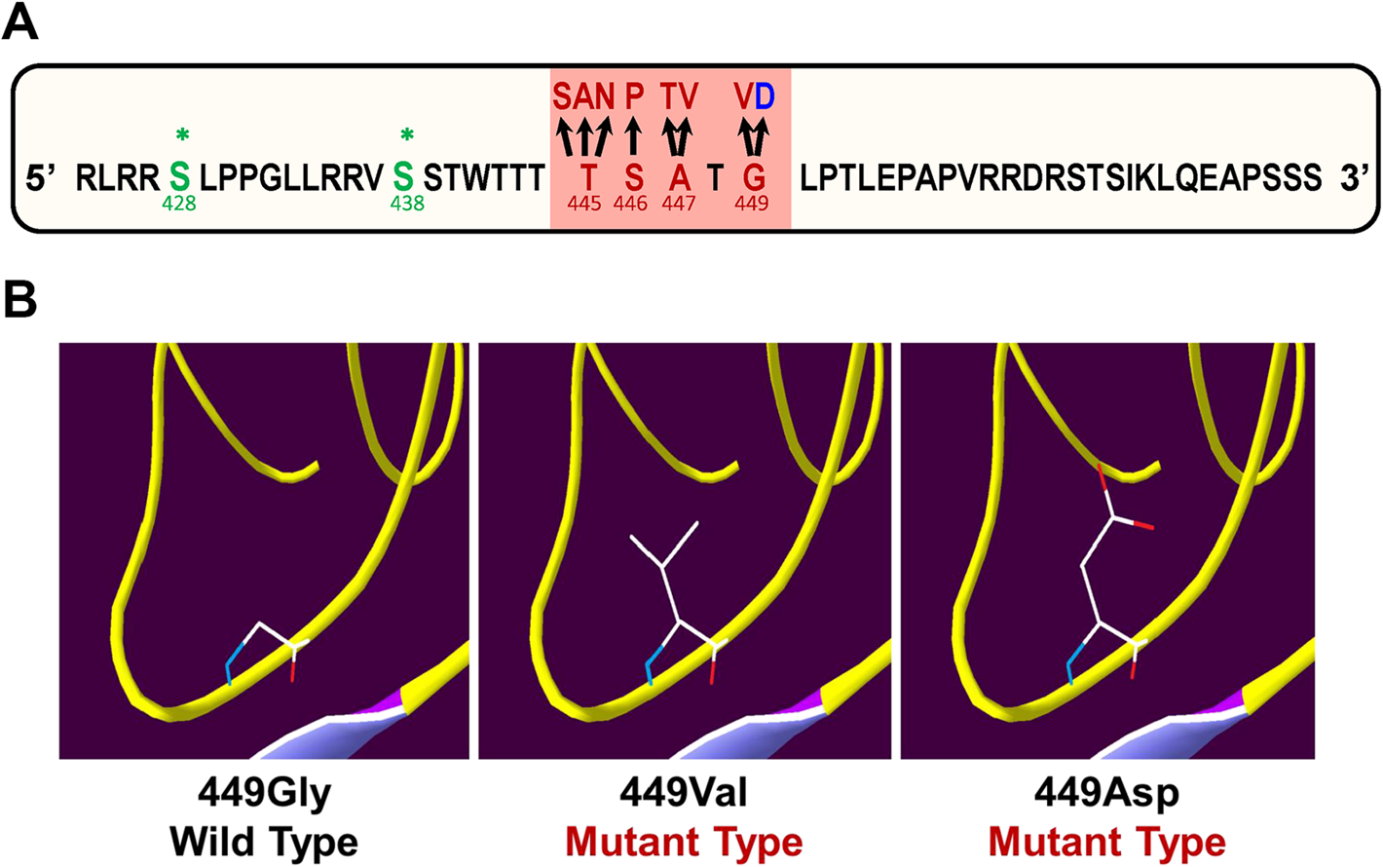
Functional analysis of the pathogenic variant *PDE3A*-p.Gly449Asp. **a** The peptide sequence of *PDE3A* exon 4, which harbours the pathogenic variant cluster (red-coloured region), and the phosphorylation sites Ser428 and Ser438 (shown in a green colour) near the altered residues are shown. Reported pathogenic variants causing autosomal dominant hypertension with brachydactyly type E syndrome are shown in red. Our novel pathogenic variant is shown in blue. **b** In silico structural modelling of the two variants (p.Gly449Val-reported, p.Gly449Asp-novel) compared with the wild type in the PDE3A protein

To date, nine pathogenic variants (c.1333A > G, p.Thr445Ala; c.1334C > G, p.Thr445Ser; c.1334C > A, p.Thr445Asn; c.1336 T > C, p.Ser446Pro; c.1339G > A, p.Ala447Thr; c.1340C > T, p.Ala447Val; c.1333_1335de, p.Thr445del; c.1346G > T, p.Gly449Val; and c.1346G > A, p.Gly449Asp) have been reported in a highly conserved cluster region in exon 4 of the *PDE3A* gene (Fig. 3a) [3, 6]. These variants are adjacent to the Ser428 and Ser438 residues, which can be phosphorylated by protein kinase C (PKC) or protein kinase A (PKA), thereby uncovering the two cryptic sites for phosphorylation and, consequently, hyperactivating the enzyme and resulting in lower cAMP levels [3, 11].

Our variant was located in the same cluster as these reported pathogenic variants and at the same position as the c.1346G > T (p.Gly449Val) variant (Fig. 3a). In protein structural modelling, these two variants presented similar structures (Fig. 3b), which implied that the two substitutions have similar functions. Based on this finding, we postulate a similar mechanism, in which our variant uncovers the Ser428 and Ser438 cryptic sites for phosphorylation and hyperactivated PDE3A. Thus, we believe that our variant causes HTNB via a mechanism similar to that reported in previous studies [3, 11]. Therefore, milrinone is a recommended choice for such patients. Further functional studies and additional genetic validation of this variant should be performed in the future.

In conclusion, we identified a de novo *PDE3A* variant in a Han Chinese family with HTNB. Our finding enriches the pathogenic spectrum of *PDE3A*-related hypertension and will facilitate future genetic diagnosis.

## Supplementary information

**Additional file 1: Figure S1.** Illustration for the filtering process of WES. (PPTX 39 kb)

## Data Availability

The hg19/GRCh37 human reference genome (https://genome.ucsc.edu/cgi-bin/hgTracks?db=hg19&position=lastDbPos) was used as the reference dataset in this study. The datasets generated and/or analysed during the current study are available in the SRA - NCBI repository, the Sequence Read Archive (SRA) accession number is: SRR11974541 (https://www.ncbi.nlm.nih.gov/sra/?term=SRR11974541).

## Abbreviations

HTNB: Hypertension with brachydactyly type E syndrome
WES: Whole-exome sequencing
CNV: Copy number variation
MAF: Minor allele frequency
