# Supplementary material for: Whole-exome sequencing identifies a de novo PDE3A variant causing autosomal dominant hypertension with brachydactyly type E syndrome: a case report
Source: BMC Med Genet. 2020 Jul 6;21:144. doi: 10.1186/s12881-020-01077-z (PMC7336660; doi:10.1186/s12881-020-01077-z)
Supplement: Supplementary file 1 — Additional file 1: Figure S1. Illustration for the filtering process of WES. (PPTX 39 kb) [file 12881_2020_1077_MOESM1_ESM.pptx]

## Slide 1
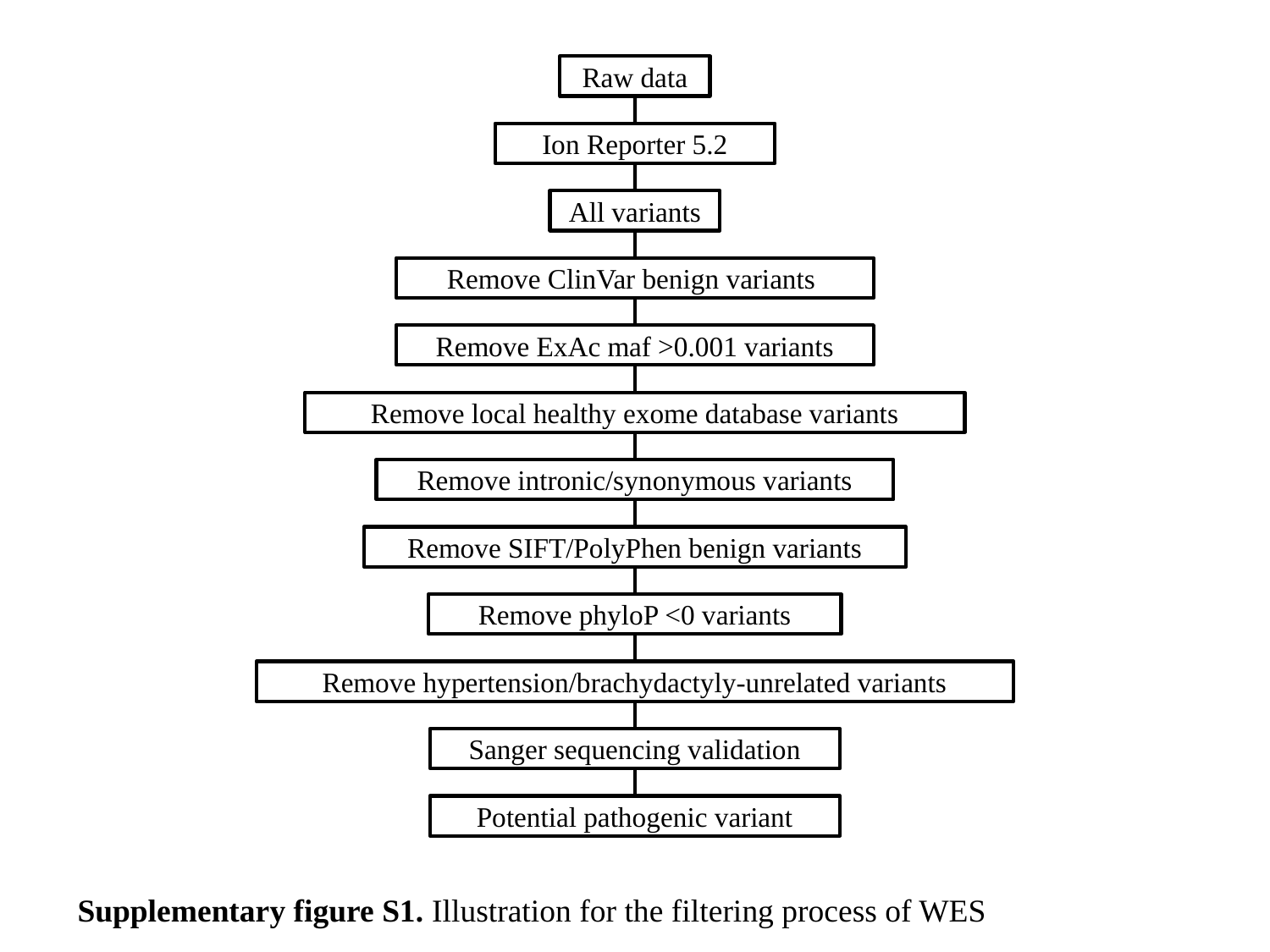

Raw data
Ion Reporter 5.2
All variants
Remove ClinVar benign variants
Remove ExAc maf >0.001 variants
Remove local healthy exome database variants
Remove intronic/synonymous variants
Remove SIFT/PolyPhen benign variants
Remove phyloP <0 variants
Remove hypertension/brachydactyly-unrelated variants
Sanger sequencing validation
Potential pathogenic variant
Supplementary figure S1. Illustration for the filtering process of WES
